# Formamidinium Lead Iodide Perovskite Thin Films Formed by Two-Step Sequential Method: Solvent–Morphology Relationship

**DOI:** 10.3390/ma16031049

**Published:** 2023-01-25

**Authors:** Věra Cimrová, Mariem Guesmi, Sangwon Eom, Youngjong Kang, Drahomír Výprachtický

**Affiliations:** 1 Institute of Macromolecular Chemistry, Czech Academy of Sciences, Heyrovského nám. 2, 162 00 Prague 6, Czech Republic; 2 Department of Chemistry, Hanyang University, Seoul 04763, Republic of Korea; 3 Institute of Nano Science and Technology, Hanyang University, Seoul 04763, Republic of Korea; 4 Research Institute for Natural Sciences, Hanyang University, Seoul 04763, Republic of Korea

**Keywords:** perovskite, formamidinium lead iodide, morphology, XRD, SEM, photophysics

## Abstract

Thin films made of formamidinium lead iodide (FAPbI_3_) perovskites prepared by a two-step sequential deposition method using various solvents for formamidinium iodide (FAI) - isopropanol, *n*-butanol and *tert*-butanol, were studied with the aim of finding a correlation between morphology and solvent properties to improve film quality. They were characterized by X-ray diffraction (XRD) and scanning electron microscopy (SEM) and their photophysical properties were studied by means of absorption and photoluminescence (PL) spectroscopies. XRD patterns, absorption and PL spectra proved α-phase formation for all selected solvents. An excessive amount of PbI_2_ found in perovskite films prepared with *n*-butanol indicates incomplete conversion. Thin film morphology, such as grain and crystallite size, depended on the solvent. Using *tert*-butanol, thin films with a very large grain size of up to several micrometers and with preferred crystallite orientation were fabricated. The grain size increased as follows: 0.2–0.5, 0.2–1 and 2–5 µm for isopropanol, *n*-butanol and *tert*-butanol, respectively. A correlation between the grain size and viscosity, electric permittivity and polarizability of the solvent could be considered. Our results, including fabrication of perovskite films with large grains and fewer grain boundaries, are important and of interest for many optoelectronic applications.

## 1. Introduction

Organic-inorganic perovskites have emerged as a highly promising class of semiconductor for optoelectronic device applications, such as solar cells [1,2,3,4,5,6,7], light-emitting diodes (LEDs) [8,9,10,11] and photodetectors [12,13,14,15] due to their many advantages including their low-cost, facile synthesis, solution processability, high absorption coefficient in the visible region, direct bandgap, ease of bandgap tuning and high luminescence quantum yield [16]. The power conversion efficiency (PCE) of perovskite solar cells was raised to 25.5% [7] and the external quantum efficiencies of LEDs based on perovskites exceeded 20% [11]. They have also shown potential in non-linear photonics [17] and as stimuli-responsive materials for a range of important technologies such as smart windows, memory devices, data storage and sensors [18,19,20,21]. Three-dimensional (3D) perovskite structures are formed by three primary ions ABX_3_ (A = Cs^+^, (CH_3_NH_3_)^+^ (MA), [HC(NH_2_)_2_]^+^ (FA); B = Pb^2+^, Ge^2+^, Sn^2+^; X = Cl^−^, Br^−^, I^−^). The A and B cations coordinate with 12 and 6 X anions, respectively, forming cub-octahedral and octahedral structures. In general, stable 3D perovskite structures are formed when the tolerance factor is within the range of 0.76–1.13, while other perovskite-related structures are stable outside this range [22]. The prototype methylammonium lead iodide (MAPbI_3_) perovskite and the mixed halide MAPb(I_1−x_Br_x_)_3_ and MAPb(I_1−x_Cl_x_)_3_ analogues have dominated the field of organometallic halide perovskite materials. In addition to MA, FA was used to provide perovskite with a smaller bandgap (~1.5 eV). Formamidinium lead iodide (FAPbI_3_) perovskite shows great advantages in photovoltaic applications not only due to its ideal bandgap energy, but also due to better charge transport properties and better environmental stability, since the instability of MA-based perovskite is partly due to the hydrophilic nature of MA [23]. The difference in ionic radius of the MA (1.8 Å) and FA (1.9–2.2 Å) ions leads to structural and optoelectrical differences between MAPbI_3_ and FAPbI_3_. It was shown that MAPbI_3_ displays an *n*-type and FAPbI_3_ a *p*-type character [24,25]. The hole-diffusion length (~90 nm) in MAPbI_3_ is shorter than its electron-diffusion length (~130 nm) [26], while the hole-diffusion length in FAPbI_3_ (~813 nm) is 4.6 times larger than its electron-diffusion length (~177 nm) [27].

FAPbI_3_ has four main different crystal structures. The common crystal forms are perovskite *α*-FAPbI_3_ and non-perovskite *δ*-FAPbI_3_ phase. Distortions by the presence of [PbI_6_]^4−^ octahedra promote the formation of lowered-symmetry *β*-FAPbI_3_ and *γ*-FAPbI_3_ black phases as the temperature decreases [28,29]. Pure α-FAPbI_3_ phase, as a very promising material for optoelectronic devices, is of great interest in the research field of perovskites. The performance of FAPbI_3_ devices can be considerably improved by stabilizing the *α*-FAPbI_3_ phase, eliminating lattice stress, improving the crystallinity and optimizing the device architecture [24,30]. Improvement of the film morphology of perovskite, i.e., fabrication of film with larger grains and fewer grain boundaries, is an important issue in optoelectronic applications, including photovoltaic devices.

One-step spin coating [31,32] and two-step sequential deposition [33,34,35,36,37,38,39] methods are commonly utilized for thin film preparation [40]. Anti-solvents are used to tune the nucleation and crystal/grain growth of perovskite precursor films in the one-step method. The film and conversion quality are influenced by anti-solvent delay dripping time. The anti-solvent treatment improves the surface morphology and promotes the crystal growth of perovskite films depending on their boiling point and polarity in relation to volatility and low solubility of perovskites compared to strong polar solvents [41]. Although the one-step method is very simple for thin film preparation, there is a problem with uneven surface coverage affecting the uniformity of the resulting film due to the uncontrollable rate of film crystallization. Various solvent and annealing engineering strategies have been developed to improve thin film quality and large grain formation [42,43,44,45,46,47]. The two-step sequential deposition of perovskite is an alternative route to one-step deposition, providing an efficient low-cost route to high performance perovskite films for optoelectronic applications. The advantage of the two-step method is easy fabrication of good-quality, pinhole-free thin films and better repeatability/reproducibility compared with the one-step method [48]. In this case, individual precursor layers are deposited separately and react with each other due to interdiffusion, forming thus a perovskite. In the sequential deposition, which was widely reported in MAPbI_3_ perovskite solar cells, two main routes were used. The first, in which a porous PbI_2_ is dipped in a MAI solution of isopropanol (IPA), leads to formation of the perovskite, where the dipping time is the critical parameter in the conversion of the perovskite phase, since interdiffusion is facilitated by the penetration of MAI into a porous PbI_2_ layer. In the second route, first PbI_2_ layer is prepared by spin coating a solution of PbI_2_ in *N*,*N*-dimethylformamide (DMF) and this is followed by spin coating a solution MAI in IPA on the top. The PbI_2_ layer has a very low solubility in IPA, therefore is not affected by the MAI spin coating. The interdiffusion method in stacked bilayers was described as different from the two-step dip coating method, as the interdiffusion reaction between precursor elements leading to perovskite is driven by thermal annealing at various temperatures for variable times, depending on the elements [40]. Recently, in-situ optical spectroscopy was used to study thin film formation of the model halide perovskite MAPbI_3_ during the spin coating of MAI on PbI_2_ in two-step coating and to understand the film formation dynamics [38]. It was found that the film formation takes place in five consecutive steps, including the initial formation of a MAPbI_3_ capping layer via an interface crystallization and the occurrence of an intense dissolution–recrystallization process leading to a fully converted temporally stable state.

In the two-step sequential method, IPA is commonly used as a solvent for the MAI (FAI). The solvents for inorganic (PbI_2_) and organic (MAI, FAI) components affect the quality and morphology of the perovskite film. There are more studies on solvent engineering concerning inorganic PbI_2_ than on organic components. As mentioned above, IPA is mostly used as solvent for organic components MAI or FAI. Cyclohexane, ethanol and cyclohexane or *n*-butanol mixed with IPA were also used as solvents for MAI or MABr [39]. *n*-butanol was used as co-solvent (2% in IPA) for preparation of MAPbI_3_ thin films possessing larger grain size (100–500 nm) for solar cells with improved PCE [49]. They attributed the improvement in the grain size to the slower solvent evaporation rate due to the addition of *n*-butanol with higher boiling point compared to IPA. Here, we have chosen IPA, *n*-butanol (*n*-BuOH) and *tert-*butanol (*t*-BuOH) as the solvents for FAI in perovskite fabrication, with the aim of revealing whether there is any correlation between the perovskite film morphology and solvent properties.

In this paper, we report on the preparation of organic-inorganic FAPbI_3_ perovskite thin films by a two-step sequential deposition method using various solvents (IPA, *n*-BuOH and *t*-BuOH) for FAI, on their characterization and on their photophysical properties. The structure and morphological properties of thin films were studied by X-ray diffraction (XRD) and scanning electron microscopy (SEM) and their photophysical properties by means of absorption and photoluminescence (PL) spectroscopies. Using *t*-BuOH, thin films with a very large grain size of up to several micrometers and preferred crystallite orientation were prepared. The obtained results are discussed in relation to the solvent properties used for FAI layer preparation. A correlation between grain size and solvent parameters is considered. To the best of our knowledge, the use of *tert*-butanol as a solvent for FAI in thin film preparation by the two-step sequential method producing FAPbI_3_ thin films with such large grains and such a correlation with the solvent properties have not been reported before.

## 2. Materials and Methods

### 2.1. Materials and Layer Preparation

Formamidinium acetate, hydroiodic acid and solvents were purchased from commercial suppliers (TCI Europe, N.V., Zwijndrecht, Belgium, VWR International s.r.o., Stříbrná Skalice, Czech Republic, Lach-Ner, Ltd., Neratovice, Czech Republic).

Formamidinium iodide (FAI) was synthesized analogously to the literature [50,51,52,53] by the following procedure. Formamidinium acetate (TCI, 11.5 g, 0.11 mol) was dissolved in ethanol (80 mL) and the hydroiodic acid (57%, TCI, 30 mL, 0.227 mol HI) was slowly added. The reaction mixture was stirred at 50 °C for 2 h, then the solvents were vacuum evaporated and the residue was worked up with chloroform. The yellow crystals were filtered off, washed with chloroform (3 × 30 mL) and crystallized from ethanol/diethyl ether. Yield: 14.26 g of FAI (75%).

Lead iodide (PbI_2_) was prepared as follows. Lead nitrate (Pb(NO_3_)_2_, 2.42 g) dissolved in hot water (750 mL) and potassium iodide (KI, 2.42 g) dissolved in hot water (750 mL) were mixed in a 2L-Erlenmeyer flask and the mixture was heated (water bath) to obtain a clear solution. Then the flask was left for crystallization in a fridge overnight. The yellow precipitate of the PbI_2_ was filtered off (S2), washed with cold water to remove easily soluble KNO_3_ and dried (oil pump). Yield: 2.70 g (80%).

FAPbI_3_ thin film preparation was performed in the glove box (M. Braun Inertgas-Systeme GmbH, Garsching, Germany) under a nitrogen atmosphere in two steps by spin-coating. In the first step, the PbI_2_ dissolved in DMF (0.46 g mL^−1^, stirring at 70 °C overnight) was spin-coated at 3000 rpm for 30 s onto the fused silica substrates (UV-ozone for 15 min before the spin coating) and then annealed at 70 °C for 10 min. In the second step, the FAI (50 mg mL^−1^) dissolved in various solvents (IPA, *n*-BuOH and *t*-BuOH) was spin-coated at 1500–2500 rpm for 15 s on the top of the PbI_2_ layer and subsequently annealed at 170 °C for 10 min.

### 2.2. Methods

X-ray diffraction (XRD) measurements were performed in theta-2theta geometry from 2*θ* = 5°–45° with 0.01° step with Cu Kα radiation (1.54 Å) using an Explorer X-ray diffractometer (GNR Analytical Instruments, Novara, Italy). The beam was collimated using Soller slits and monochromatized by Ni filter and Mythen 1k Strip detector (CeleriX, Baden-Daettwil, Switzerland) with an active area of 62 mm × 8 mm and 1280 tailorable pixels. The sample−detector distance was 239.6 mm. A high-resolution FE-SEM (JEOL Ltd., Tokyo, Japan) JSM-7800F Prime (resolution: 0.7 nm at 15 kV) equipped with an in-lens Schottky plus field emission electron gun was used for the thin-film morphology characterization. A thin conductive layer of Pt with a thickness of ~20 Å was deposited on the films before SEM. Software VESTA was used for the structural model visualization [54]. UV–Vis spectra were measured on a Perkin-Elmer Lambda 35 UV/VIS spectrometer (PerkinElmer Instruments, Shelton, CT, USA). Photoluminescent (PL) spectra were recorded using a Perkin-Elmer LS55 Fluorescence spectrophotometer. Layer thicknesses (250–300 nm) were measured using a KLA-Tencor P-10 profilometer (KLA-Tencor Corporation, Milpitas, CA, USA). The measurements were performed at ambient atmosphere.

## 3. Results and Discussion

**Figure 1:**
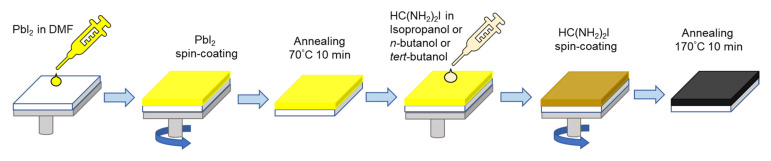
Scheme of two-step sequential deposition method of FAPbI_3_.

Thin FAPbI_3_ perovskite films were prepared by a two-step sequential deposition method, which is schematically shown in Figure 1. In the first step, the PbI_2_ layer was spin-coated from the DMF and in the second step FAI in various solvents (IPA, *n*-BuOH and *t*-BuOH) was used for the spin-coating process. After thin film annealing at 170 °C for 10 min, black FAPbI_3_ *α*-phase was obtained, which was confirmed by XRD measurements.

**Figure 2:**
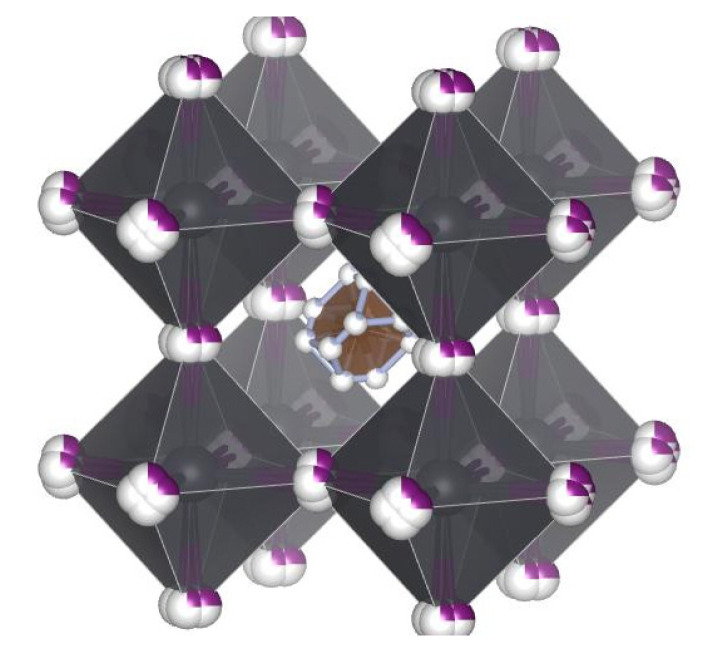
Schematic view of perovskite cubic structure and structural model of FAPbI_3_ (FA cation located in the middle).

As already mentioned in the Introduction, the crystal structure of FAPbI_3_ is polymorphic due to the large ionic radius of FA. The structural diversity and phase behaviours of the FAPbI_3_ are related to the orientation of the FA cation and the degree of the crystal lattice distortion. In some of the literature, the *α*-FAPbI_3_ adopted a trigonal phase corresponding to *P*3*m*1 space group [25,55,56,57,58,59], but based on the crystal structure of α-FAPbI_3_ determined by high-resolution neutron powder diffraction, which is much more sensitive for the identification of light atom positions than XRD, it was shown to adopt a simpler cubic phase with P*m*$\bar{3}$*m* symmetry [60]. In the *α*-FAPbI_3_ crystal, the C–H in FA cation pointed directly toward the cube’s face and the FA cations with high rotational mobility are dynamically disordered within the octahedron framework due to the potential formation of hydrogen bonds between –NH_2_ and I [60,61]. A schematic view of the perovskite cubic structure and the structural model of FAPbI_3_ is shown in Figure 2.

**Figure 3:**
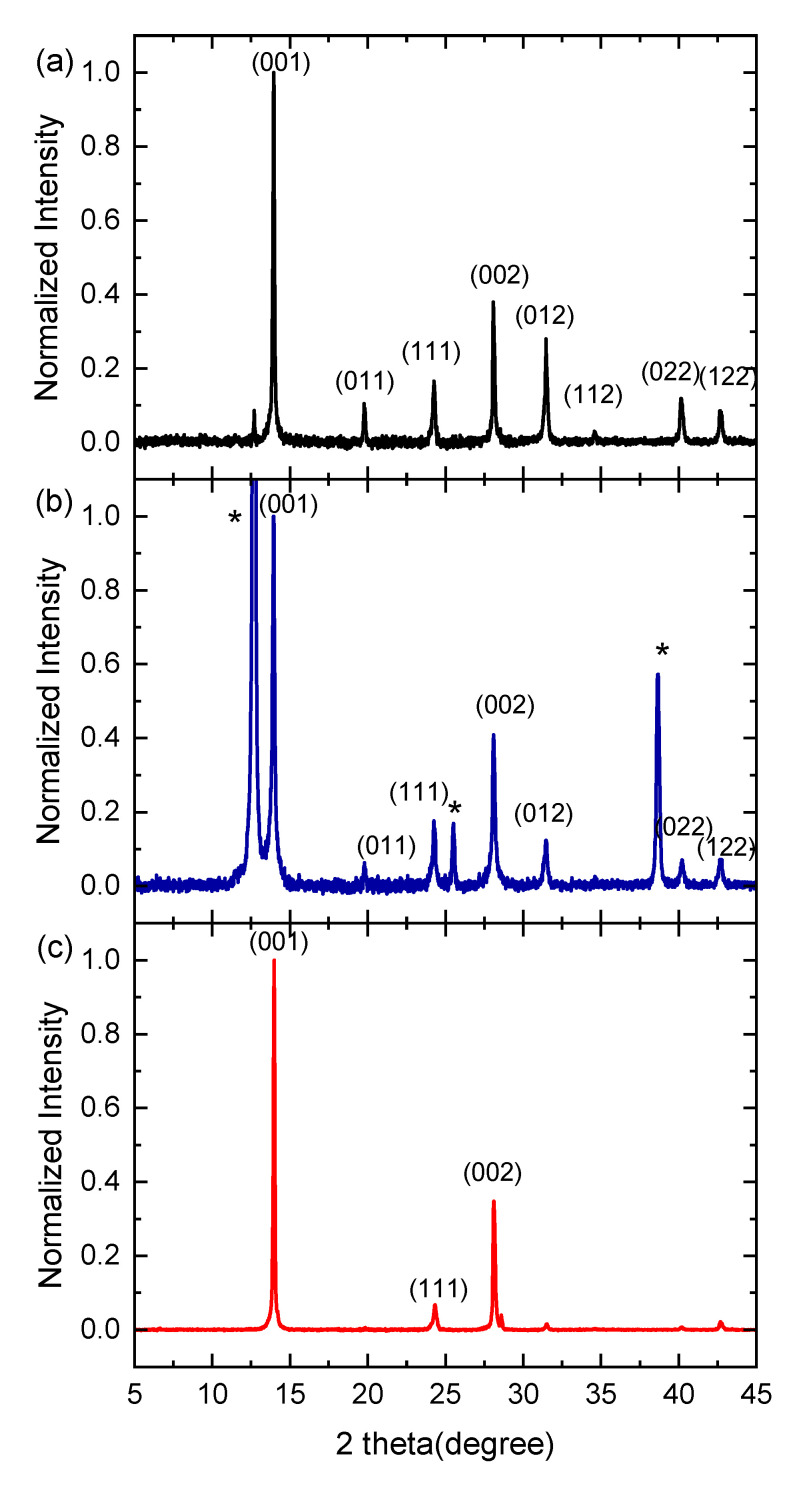
Normalized X-ray (XRD) patterns of the thin film perovskite FAPbI_3_ prepared on fused silica by two-step method using various solvents for FAI: (**a**) IPA, (**b**) *n*-BuOH and (**c**) *t-*BuOH (* correspond to PbI_2_).

X-ray patterns normalized to the dominant perovskite peak of FAPbI_3_ are displayed in Figure 3. The typical reflection peaks for FAPbI_3_ *α*-phase were identified for all films prepared using the selected solvents for FAI. XRD patterns of our films were verified as a single cubic phase, consistent with the literature [29,62,63,64,65,66,67,68]. XRD pattern of the films prepared with isopropanol shows peaks at 13.94°, 19.79°, 24.26°, 28.07°, 31.45°, 34.55°, 40.14° and 42.63° assigned to the reflections of the (001), (011), (111), (002), (012), (112), (022) and (122) crystal planes of cubic perovskite α*-*FAPbI_3_ structure, respectively. In the XRD pattern of FAPbI_3_ films prepared using *n*-BuOH as a solvent for FAI, the peaks at 12.7°, 25.5° and 38.7° appeared in addition to the perovskite peaks. These peaks (* in Figure 3b) with the dominating one at 12.7° correspond to PbI_2_ and indicate an incomplete perovskite conversion, related to the PbI_2_ excess. The PbI_2_ deposited from a DMF solution was crystallized as a hexagonal 2H polytype. The appearance of only three diffraction peaks corresponding to (001), (002) and (003) in XRD pattern lattice planes indicates the growth orientation along the *c* axis [40].

The XRD pattern for the FAPbI_3_ film prepared from *t*-BuOH exhibits two major (001) and (002) diffraction peaks at 13.97° and 28.1°, respectively. This observation indicated preferable growth along (001) and (002) crystallographic planes. In addition, a slight shift of the (001), (111) and (002) peaks to higher 2θ values in the films compared with powder is likely due to the possible shrinking of the crystal lattice in the film. The lattice parameter values (a) given in Table 1 were evaluated using the Bragg relation n λ=2 dsinθ, where *n* is the order of the interference, λ is the X-ray wavelength, θ is the angle of incidence, *d* is the lattice plane spacing and the relation for the cubic crystal structure

$$\frac{1}{d^{2}}=\frac{h^{2}+k^{2}+l^{2}}{a^{2}}$$

where *h*, *k*, *l* are the Miller indexes. Compared with the powder value of 0.6362 nm [60] the lower values in the range of 0.6342–0.6351 nm were obtained for all films, which correlate well with the average lattice parameter of 0.6335 nm obtained from the density functional theory (DFT) calculations [69].

Obvious differences in the ratios of peak intensity were found for the films prepared from different solvents, which indicates the influence of solvent on crystallinity and crystal orientation. The absolute intensity of both major peaks at 13.97° and 28.10° increased for the films prepared with *t-*BuOH, which shows the better crystallinity of these films than that of the films prepared with IPA or *n-*BuOH. A high degree of crystallinity is useful for optoelectronic applications because it facilitates charge carrier transport [59].

The XRD technique is a useful tool for determination of the crystallite size from the line broadening [70,71,72]. A primary source of peak broadening is related to the crystal domain size. The larger crystallites enhance intensity, while the smaller crystallites merge in the base of the peak. The relationship between peak full width at half maximum (FWHM) and the average crystallite size is given by the equation suggested by Scherrer in 1918 [73]

(1) $$D=\frac{K\lambda }{\beta_{hkl}\cos\theta }$$

where λ is the X-ray wavelength, *D* the average crystallite size, $\beta_{hkl}$ the FWHM of the reflection peak located at angle 2θ after subtraction of the instrumental broadening ($\beta_{hkl}=\sqrt{\beta_{measured}^{2}-\beta_{intrumental}^{2}}$), and *K* the Scherrer constant is a dimensionless shape factor. The value of *K* depends on the crystallite shape and the crystallite-size distribution and was found to be in the range of 0.62–2.08 [74]. There is uncertainty in *K* if the shape and distribution of crystallites are not known. The *K* value is typically at about 0.9. In our evaluation, we have used the value of 0.94, which is used for spherical crystallites with cubic crystal symmetry [70]. This method is applicable for the determination of the crystallite size of the order of 0.1 µm and appropriate for stress-free samples.

It should be noted that for thin films there are other sources of peak broadening that need to be considered. Thin-film materials are prone to maintain a certain strain in the lattice, which affects their average structure and shifts the peak positions (macrostrain); whereas the strain at a microscopic level (microstrain) affects the peak broadening [70]. There is strain induced in the sample due to crystal imperfection and distortion and distribution of strain within crystallites from the boundary to the core. We calculated the microstrain in the films by analysing the FWHM in the diffraction patterns according to the Williamson Hall (WH) plot method proposed by Williamson and Hall in 1953 [66]. This method is currently widely used for microstrain and crystallite size analysis for thin film polycrystalline samples to separate the crystallite size and strain contribution from XRD peak broadening. The relationship between the peak width $\beta_{\varepsilon }$ and microstrain ε is given by the Wilson equation [70]:

(2) $$\beta_{\varepsilon }=4\varepsilon \tan\theta$$

Combining Equations (1) and (2), the microstrain ε and crystallite size *D* in the films can be determined using the equation:

(3) $$\beta_{hkl}\cos\theta =\frac{K\lambda }{D}+4\varepsilon \sin\theta$$

The slope and intercept of the straight line of the plot $\beta_{hkl}\cos\theta \;\mathrm{vs}.\;\sin\theta$ give the value of microstrain and average crystallite size, respectively. The major peaks were used for evaluation. Very low microstrain values (<0.03%) were evaluated for all FAPbI_3_ films independent of the solvents used. The crystallite sizes are listed in Table 1. It was found that the solvent also influenced the crystallite size. The largest crystallite sizes are in the films prepared with *t-*BuOH and the smallest for the films prepared with *n-*BuOH, which possesses a higher boiling point and lower vapor pressure than the other two solvents.

**Figure 4:**
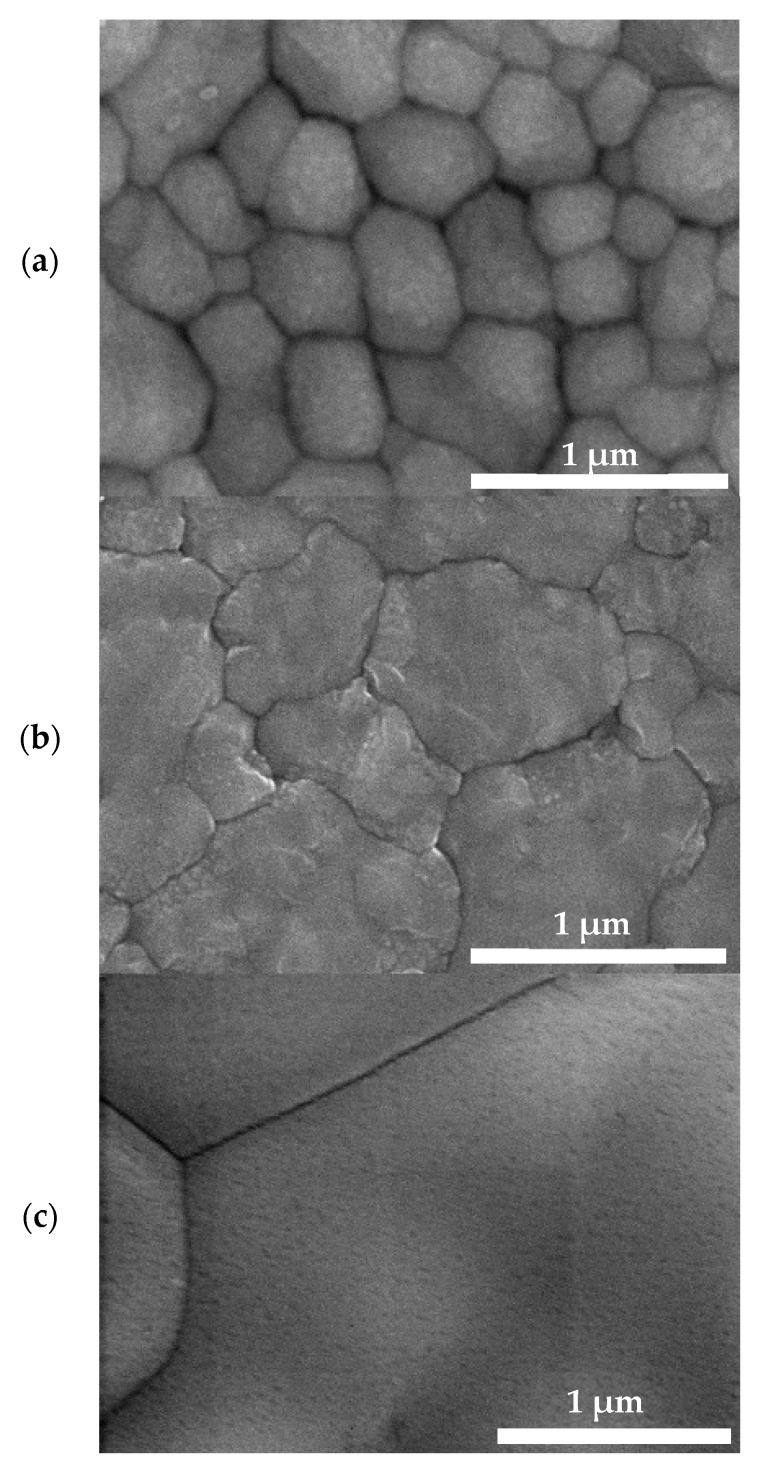
SEM images of the thin film perovskite FAPbI_3_ prepared by two-step method using various solvents for FAI: (**a**) IPA, (**b**) *n*-BuOH and (**c**) *t-*BuOH.

The surface morphology was studied by SEM to compare the morphology of the thin films and to evaluate the size of grains in relation to the solvent used. Figure 4 shows SEM images of the FAPbI_3_ films. Larger grains were found in the films prepared using the *n*-BuOH and *t-*BuOH as a solvent for FAI than in the films where IPA was used (see Table 1). Very large grains of 2–5 µm were found in films prepared with *t-*BuOH. The advantage of large-size crystallites, grains and limitation of grain boundaries is that these usually lead to efficient charge carrier transport and hence to the improvement of optoelectronic devices. We tried to correlate the morphological data, grain and crystallite sizes with the properties of the solvents used for FAI spin-coating. Selected properties of the solvents, which were taken from the literature [75], are listed in Table 2. For the solvents under study, a correlation between the grain size and viscosity (*µ*), electric permittivity (*ε*_r_) and polarizability (*α*) of the solvent could be considered. The grain size increased with increasing solvent viscosity and polarizability and with decreasing solvent electric permittivity. On the other hand, crystallite size could be considered in relation to the boiling point (*T*_bp_) and vapor pressure(*p*_v_). Larger crystallite sizes were evaluated for the films prepared with IPA and *t-*BuOH, i.e., solvents having similar boiling point and vapor pressure, whereas the crystallite size was smaller for the films prepared using *n*-BuOH with a higher boiling point *T*_bp_ and lower vapor pressure *p*_v_.

Our findings could be correlated with description of the film formation as mentioned in the Introduction, reported for MAPbI_3_ film analyzed during preparation by two step spin-coating [38]. Analogously, the following steps can also be considered for the FAPbI_3_ film formation during FAI spin-coating on the top of PbI_2_ layer. In the first step, the FAPbI_3_ capping layer is formed on the top of PbI_2_, which prevents further FAPbI_3_ crystallization. Therefore, in the second step, the FAI solution concentration above the capping layer increases due to solvent evaporation. In the third step, the FAPbI_3_ capping layer begins to dissolve due to the increased iodine I^-^ concentration as a result of progressing solvent evaporation. The fourth step involves a rapid main dissolution–recrystallization process, in which lead iodine complex Pb_4_^2−^ (formed by the reaction of PbI_2_ and 2I^−^ with the increasing concentration) reacts with FA cation leading to the FAPbI_3_ formation. The dissolution process of the capping layer generally occurs at the grain boundary and smaller grains, where new crystals are formed and increase the grain size. One can assume that the process is slower with increasing viscosity, which can facilitate larger grain formation. Finally, the complete fully converted and temporally stable state is formed in the fifth step.

**Figure 5:**
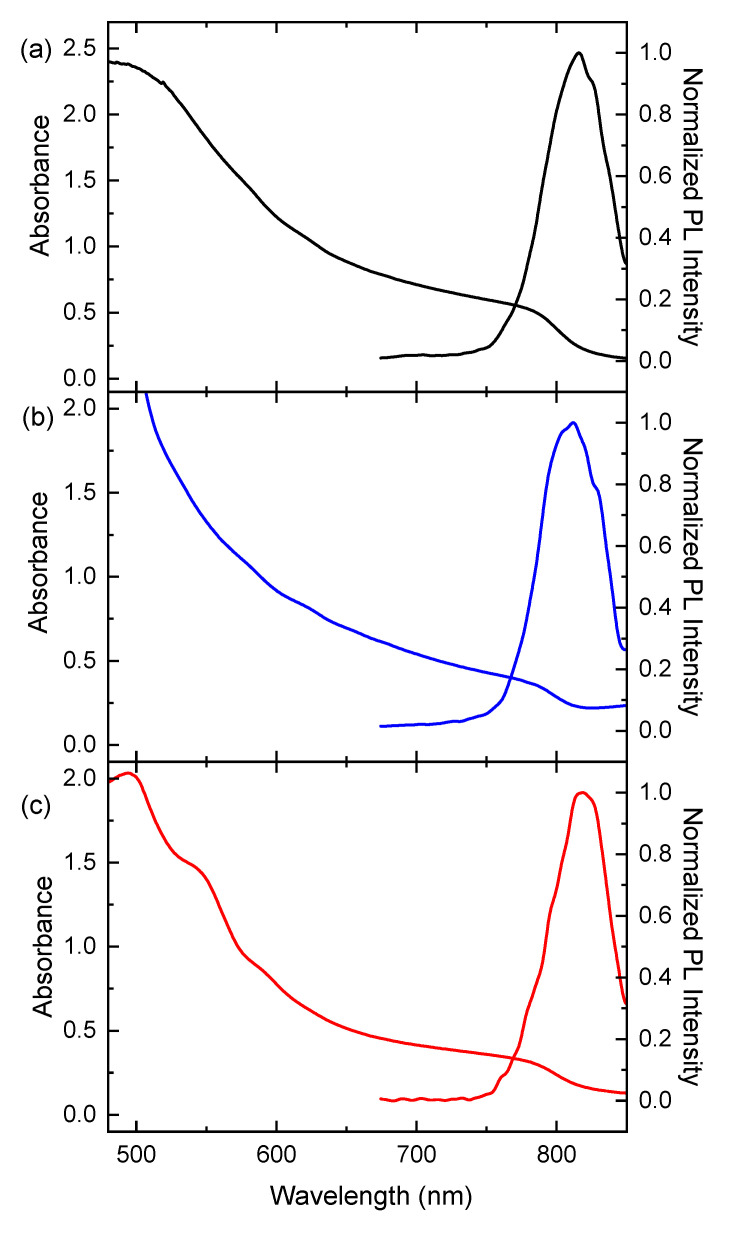
Absorption and PL spectra of the thin film perovskite FAPbI_3_ prepared by a two-step sequential deposition method using various solvents for FAI: (**a**) IPA, (**b**) *n*-BuOH and (**c**) *t-*BuOH.

Absorption and PL spectra have also confirmed FAPbI_3_ *α*-phase structure. Examples of absorption and PL spectra of the thin films are displayed in Figure 5 and photophysical data are summarized in Table 1.

The shapes of absorption spectra are typical for 3D black *α*-phase FAPbI_3_. Absorption coefficient values (in the range of 1.4–1.8 × 10^7^ m^−1^ at 500 nm, 6.6–7.5 × 10^6^ m^−1^ at 600 nm and 3.7–4.3 × 10^6^ m^−1^ at 700 nm) also correspond to those reported for this phase [69,76]. In the range of wavelength from 500 to 700 nm, the penetration depth values are from 55 to 270 nm. The bandgap (*E*_g_) values evaluated from the Tauc plot (α*E)^2^ vs. *E* are about 1.5 eV, similar for all films prepared using IPA, *n*- and *t*-BuOH. They are in good agreement with the values reported for FAPbI_3_ thin films in the range of 1.46–1.53 eV [27,56,59,76,77] and are also consistent with the reported theoretical values from 1.44 to 1.6 eV [69,78,79]. PL peaks correspond well to the absorption onsets, which suggests that the observed photoluminescence is predominantly from the bandgap.

## 4. Conclusions

Morphology and photophysical properties of FAPbI_3_ thin films prepared by a two-step sequential deposition method using three different solvents for FAI (IPA, *n*-BuOH and *t-*BuOH) were studied. The morphology of thin films differed and was correlated with the selected solvent properties. A correlation between grain size and solvent viscosity, electric permeability and polarizability was found. We succeeded in the preparation of *α*-FAPbI_3_ thin films with large grains up to several micrometers with preferred crystallite orientation using *t-*BuOH as solvent for FAI. Grain size increased for the solvents as follows: IPA < *n*-BuOH < *t-*BuOH. This correlates with increasing solvent viscosity and polarizability and decreasing electric permittivity. In FAPbI_3_ films prepared using *n*-BuOH as a solvent for FAI, an excessive amount of PbI_2_ was found, which indicated an incomplete perovskite conversion. The evaluated crystallite sizes were largest for the FAPbI_3_ films prepared using *t-*BuOH, then with IPA, and the smallest sizes were evaluated for the films prepared with *n*-BuOH possessing higher boiling point and lower vapor pressure. The microstrain value was determined to be lower than 0.03% for all films under study. All FAPbI_3_ thin films exhibited a bandgap of about 1.5 eV and photoluminescence with maxima at 810–820 nm corresponding to the bandgap luminescence regardless of the solvent used for FAI. Our study provides insight into FAPbI_3_ thin film fabrication with large grains by two-step sequential deposition given by choice of solvent, which could be important for many optoelectronic applications.

## Figures and Tables

**Table 1:** Morphological and photophysical data (*a* is lattice parameter, *D* crystallite size, *L* grain size, *E*_g_ bandgap, *λ*_PLmax_ maximum of photoluminescence).

Solvent	*a* (nm)	*D* (nm)	*L* (µm)	*E*_g_ (eV)	*λ*_PLmax_ (nm)
IPA	0.6351	66–80	0.2–0.5	1.510	816
*n*-BuOH	0.6346	55–65	0.2–1	1.504	812
*t-*BuOH	0.6342	93–99	2–5	1.503	818

**Table 2:** Selected properties of the solvents used for FAI (*M*_w_—molar mass, *ρ*—density, *T*_bp_—boiling point, *ρ*_v_—vapor density, *p***_v_**—vapor pressure, *µ*—viscosity, *RP*—relative polarity, *ε*_r_—electric permittivity, *µ*_d_—dipole moment, *α*—polarizability).

Solvent	*M*_w_	*ρ* (g/mL) at 25 °C	*T*_bp_ (°C)	*ρ*_v_ (vs. Air)	*p*_v_ (hPa) at 20 °C	*µ* (10^−3^ Pa s)	*RP*	*ε*_r_	*µ*_d_ (D)	*α* (Å^3^)
IPA	60.1	0.785	82.4	2.1	44	2.07	0.546	19	1.66	6.98
*n*-BuOH	74.12	0.81	117.6	2.55	6.3	2.59	0.586	17.5	1.7	8.79
*t-*BuOH	74.12	0.775	82.2	2.55	41	3.35	0.389	12.4	1.7	8.82

## Data Availability

Not applicable.
